# Mechanisms for Interpretative Cooperation: Fan Theories in Virtual Communities

**DOI:** 10.3389/fpsyg.2021.699976

**Published:** 2021-06-10

**Authors:** José Manuel de Amo, Anastasio García-Roca

**Affiliations:** Department of Education, University of Almería, Almería, Spain

**Keywords:** digital reading, transmedia, fan theories, interpretation, virtual community, informal learning, literacy

## Abstract

This work focuses on analyzing fan theories as interpretive processes shared by users of virtual reading communities. Within these spaces of participatory culture, a complex strategy of interactions among its members is encouraged to formulate conjectures about the text's intentions and negotiate its degree of relevance. We have based our research on a methodology linked to the ethnography of reading. From a representative sample of the narrative universes of A Song of Ice and Fire and Harry Potter, the different modes of agreed collaborative textual interpretation are explored. The data show that within these communities of practice, three reading models are developed: predictive theories in which future narrative contents are inferred; explanatory theories in which narrative arcs are endowed and charged with meaning through the analysis of canon and, finally, alternative theories with a highly creative component in which the interpretative limits of the text are explored. Within these virtual communities, hermeneutical proposals are characterized by the activation of complex, heavily referenced literary argumentation to maintain semiosis active and expand the horizon of expectations of its members.

## Introduction

Numerous studies show how technological revolutions are changing the modes and habits of reading (Landow, 2009; Naseri and Noruzi, 2016; Cruces, 2017; Chartier, 2018; Amo, 2019). In addition to the reception of a hypertext, focused on hyperconnected fragments, the Internet has made the act of reading cease to be conceived exclusively as a solitary or individual cognitive activity and acquire a more social dimension: reading becomes conversation (Cordón, 2016; Lluch, 2017, 2018; Rovira-Collado, 2017). Thus, affinity spaces are created so members can share hobbies, objectives, and interests around books, authors, or themes (Gee and Hayes, 2011). They also collaborate to develop complex negotiations of meaning and sustain the collective semiosis of the text. Within these flexible and decentralized interactive structures, reading becomes linked to writing (the read/write culture). Readers not only activate their knowledge about code conventions (literary, audiovisual, or hypermedia), generate reading hypotheses, establish connections, and either confirm or frustrate their expectations (Culler, 2004: p. 80), but also play a more dynamic and creative role that transcends textual interpretation (from the canon), becoming content generators or protagonists in the circulation of meaning.

In these scenarios, there is a particular type of participatory commitment to the text and with other users, since belonging and recognition within the group, as well as its fascination with the source work, enhance the motivation to develop the social value of reading and the various literacy manifestations (Paladines-Paredes and Margallo, 2020). Fandom, from this perspective, is a committed, critical and intertextual practice (Gray et al., 2007). Fandom users behave as an authentic collective intelligence (Lévy, 2004; Jenkins et al., 2015) in which the interpretation of the text (fanon) together with promoting and guiding the creation of hypertextual manifestations is jointly negotiated but does not always reach consensus. In short, these are collective strategies leading to the formation of a reading community that interprets texts beyond the guidelines set by hegemonic culture (Rovira-Collado, 2017).

In this context, transmedia narratives (hereafter, TN) are particularly relevant (Jenkins, 2009). They are defined as complex semiotic systems that extend to different media, platforms, or formats and demand from their audience the activation of more sophisticated and complex reception mechanisms than in traditional reading. In an attempt to synthesize this dense concept, Scolari (2013) places its specificity in the sum of canon and fandom. The first refers to the contents that follow a top-down or descending logic and the diegetic universe that they develop (Guerrero-Pico, 2014). It is essential to clarify that they include official elements and, as described by Genette (1989, p. 316), all epitexts such as interviews with authors, marketing strategies, and official social networks (Lluch et al., 2015). Fandom, meanwhile, refers to the amateur community created around a cultural phenomenon, as well as its activities.

Important transformative or hypertextual creations are created within fandoms (*fanfics, fanfilms, remixes, fansubs*), characterized by being constantly built on and are, therefore, incomplete. Its production and reception mechanisms question the competencies traditionally attributed to the author and the reader (Fathallah, 2017). They are also part of the popular cultural and daily practices of young people (Lankshear and Knobel, 2010) and are developed in parallel with formal learning environments, linguistic and literary skills (Black, 2008; García-Roca, 2020), as well as the collaborative skills that new information and communication technologies require.

TNs develop complex fictional worlds from their possible narrative extensions into different media. In the words of Iser (1987), they intentionally play with the unsaid and encourage intersubjective discussions on individual or collective realizations of meaning and possible interpretations. Non-textual connections are made, and persuasive reasoning is made by searching for evidence or clues in corroborative works.

The creation of glossaries and other narratological elements in wikis are collaborative activities linked to collective intelligence (Booth, 2009). From this perspective, in the shared construction of community meanings and interpretation (fanon), there is often disagreement, proof of the subjective, polyphonic, and heteroglossic nature of TN (Thomas, 2018). These hermeneutic experiences have critical implications for multiliteracy and, in particular, reading training (Duncan, 2008).

Information processing is also a relevant aspect of fandom. For example, in cross-media narratives, readers of the literary saga (and other media) are often separated from those who watch films or television series. This segregation is due to the availability of different canon. The readings of members of virtual communities are also synchronized (Silva et al., 2015; García-Roca and Amo, 2019): accessing fragments of information in advance, which could ruin the surprise factor of narrative turns. Spoilers are fragments of narrative information shared among fans before the official narrative is made available (Hills, 2012). Some within the fandom focus on the creative challenge of drawing inferences and guesswork and enjoy advances, leaks, theories, and rumors (Johnson and Rosenbaum, 2017; Völcker, 2017). Mittell (2009) uses the concept of forensic fandom to describe the long-term commitment experienced by users who dissect the canon and immerse themselves in the deepest interpretative layers. He states that being a follower of these narratives implies:

to embrace a detective mentality, seeking out clues, charting patterns, and assembling evidence into narrative hypotheses and theories. This forensic engagement finds a natural home in online forums, where viewers gather to posit theories and debate interpretations, and fan wikis (Mittell, 2009, pp. 14–15).

Fan theories occupy a preferential place among fanon practices (Gray and Mittell, 2007). However, there is a lack of empirical studies that systematically and comprehensively analyze their structure, function, and definition. Prior research links fan theories, speculations, or hypotheses to forensic fandom and expand the narrative (Mittell, 2009). In addition, these theories are integrated into mechanisms for constructing shared meaning that allows readers to access more profound levels of interpretation than those achieved individually.

Fan theories are interpretative practices published within the virtual community in order that they may be discussed, modified, or ratified by the rest of its members. These are creative activities whose producers want to demonstrate to other users their reading competence (Aranda et al., 2013) or their degree of proximity to the model reader registered in the canon. This is a “lazy machine” (Eco, 1993) since the text requires the reader's participation to be potentially updated. It follows that the interpreter takes possible interpretations, and the various reading paths are foreseen and plotted by the text from the beginning since these movements form part of its own generative mechanism (Eco, 1993: p.79). It is, therefore, a textual strategy that the author must design or project. The work requires the receiver to activate the same elements of the reading competence described in the text to exhaust all possible meanings. This requisite receiver is not an empirical recipient but another mechanism of the text, built for interpretative cooperation: the Model Reader. Interpretation, therefore, necessarily implies a dialectic between the strategy of the text (model reader) and the actual reader's response (p. 86).

From this perspective, this work aims to explore the mechanisms of interpretative cooperation of TN in their natural context and, specifically, seeks to answer the following question: What role do fan theories play in the reception of narratives in which they are included? It explores the meanings that the analyzed communities confer on reading to understand the implications of reading in the configuration and functioning of the community and what rules they use in the interpretation process. From this perspective, the following operational objectives have been established:

1 Explore the modes of interpretation that are generated in virtual reading communities.
2 Analyze and describe the development and impact of interpretative processes within the fandom.
3 Classify fan theories according to the type of interpretation and their argumentative and persuasive techniques.

## Materials and Methods

Qualitative research has been carried out in which the modes of textual interpretation are addressed by analyzing the discourse of different hermeneutical proposals shared within the network. It is an ethnographic study in which specific aspects of communities are studied in their natural context to know and understand them (Hine, 2004). Based on the idea of interpretation as a social practice, it is interesting to observe the processes of communicative interaction of its members, the types of literary practices, their rules of negotiation of meaning, and their reading canon.

We have taken a sample of fan theories between 2015 and 2020 of two of the most socially vibrant fandoms linked to the TN originating from literary texts: *A Song of Ice and Fire* and *Harry Potter*. Regarding the sample selection, we have opted for the most relevant theories of the two official fandom spaces (*Pottermore* and *Westeros*) and related virtual scenarios created by fans (e.g., *lossietereinos*, wikis, or fandom communities). Texts have been analyzed until the theoretical saturation of the data and analysis categories was achieved. In the search and data collection process, the digital archive *Wayback Machine* files from *Archive.org* and the *Google* search tool were utilized to analyze fan theories at the time of their publication vis-à-vis the canon. Note that while selected narratives began more than 20 years ago, fan theories, in contrast, are usually phenomena with limited temporal vibrancy.

The data analysis has been based on a holistic and emerging coding and categorization process with the help of the *Atlas.ti* data analysis program. Despite being based on predetermined categories extracted from the bibliographic review, they have been developed and re-coded throughout the research process.

- Each fan theory has been analyzed around the following variables.
- Argumentation: the primary narrative evidence that underpins fan theories has been categorized.
- Intent and projection: we have analyzed what creative strategies are used to complete the indetermined gaps in the narrative and expand the narrative universe, that is, prediction of the end of the story, more detailed description of a narrative situation, development of motivations of secondary characters, clearing unknowns linked to the general plot, etc.
- Adaptation to the canon (as shared interpretation): in this category, the reactions of other users have been essential.
- In addition, four elements have been added that allow us to delve into the characteristics of each of the fan theories analyzed.
- Impact on the general readership and appropriation by the community of followers.
- Inclusion of insider information or spoilers.
- The obsolescence of theory is whether reading solves aspects that will soon be developed in the canon or proposed in the long term (outcome).
- Reaction from the authors and producers in the cases where the theories have been officially confirmed or refuted.

A researcher triangulation has been performed with the help of research experts with whom the mechanisms of coding, categorization, and development of hermeneutic models have been discussed and adjusted to minimize the subjective bias of the data analysis process.

## Results

Fan theories are interpretative proposals made by readers which are discussed, contrasted, and shared within affinity spaces. They anticipate or infer future content, explain specific events, or propose alternative visions. These interpretations are accompanied by solid arguments and precise references to canonical elements that give them greater likelihood and credibility: textual fragments are cited, frames are shared, or information from other media is shared as links.

Fan theories are constantly being evaluated and evolved. Some fan theories are finally confirmed, others are thwarted by the established canon. This notwithstanding, it is rare for the author's intention to prevail in these processes of interaction between the text and its readers and between the indications of the text and the readers' response. Other theories are reconfigured and end up being adapted to the canon. Examples of these include those regarding the homosexuality of Dumbledore or the prophecy of the Three Brothers, which theorizes that these characters are actually Harry Potter, Voldemort, and Dumbledore. J.K. Rowling, the original author, has confirmed these theories. Starting in 2011 on the *Westeros* forum with a simple paragraph about Cainna (2011), another theory states that Bran is the King of the Night. This theory is currently in force, although much more consolidated, having been further developed with different proposed arguments from those initially put forward. Therefore, they are loaded with content through collective intelligence with details from the most up-to-date hypotext or base text are constantly added.

Likewise, theories that question and contradict others are detected. In this sense, there is evidence of different outcomes proposed related to the Iron Throne.

Sometimes the lines that separate fan theories from fanfictions are too fuzzy: theories become so complex that they create possible and alternative lines of argument. The creativity and over-interpretation of fans interact to expand the history of hypertext (or canon) and thus the process of receiving these stories. Some theories, fully integrated into the fanon, acquire such relevance in fandom that they are not refuted, even though they may not fit with the coherence of the narrative in the subsequent canon.

The inductive analysis of the data has established three broad categories depending on the objective of the theory, the arguments used, the degree of obsolescence, their temporal location, their impact, and the textual appropriation by the members of the community. However, it is important to note that the categories are not siloed theories but instead often complement each other.

### Predictive Theories

Predictive theories aim to advance and infer possible narrative actions that will be developed in the canon. From the reception point of view, these hermeneutical proposals reflect the unconscious and individual exercise of conjecture performed during reading.

These theories are not necessarily developed by fans, that is, by users with a significant intellectual, emotional and social involvement with the narrative or cultural product, but rather, are proposed and led by a less specialized and single-media readership, that of the literary, cinematographic, or television saga. Although they generate considerable debates, they do not go beyond the surface layer of the narrative. These are individual and disconnected theories that do not undergo a clear evolution.

Predictive theories are the most numerous and decentralized: being found in non-specialized spaces such as social media networks such as *Twitter* and *Facebook* through the use of *hashtags*. As narrative hypotheses, they are ephemeral and have little transcendence for the community: any user can guess the plot's outcome without mastering all available content. In this sense, these ideas usually lack a rigorous foundation or a clear justification: they are directly linked to the outcome (the Iron Throne or Voldemort's death) or explicit interpretative gaps in history.

Readers generate their hypotheses individually and then share them on a network for discussion. Generally, during this reading process, the underlying arguments for these theories are not examined. They often rely on the latest published narrative elements, but occasionally on epitextual marketing elements such as trailers or narrative advances.

They are temporary interpretative proposals since, over time, they end up either being confirmed or refuted. It should be noted that predictions are only generated in serialized productions where there is synchronization in its reception by the reader communities. They are contextualized theories at a time in the narrative. For example, the predictive theories developed in 2006 (with *Order of the Phoenix* and *Feast of Crows* as the latter publications of these narratives) are not the same as those developed in 2012 (*Dance of Dragons* and *Deathly Hallows*). For example, new followers who have read the Harry Potter heptalogy will not raise or read these shared predictions on the Internet as they already have the outcome at their disposal.

Some notable examples of predictive theories are:

- The quest for *Valonqar*: it was speculated that it could be Jaime (and return to being the *kingslayer*), Tyrion, Jon, Danaerys, Arya, Stanis, or even Sam.
- Who would sit on the Iron Throne: the house Stark, Lanister, Targaryen, Baratheon, *The Night King*, all or none of them.
- The resurrection of Jon Snow was a consolidated and shared theory. For one part of the fandom, it was not a surprise but rather a confirmation.
- *Bran is the Night King*, which, despite being developed before the television series, gained particular relevance in the *fandom* due to the physical resemblance of the two characters. This theory has evolved in different directions, for example, in the alternative theories of Faillace (2019) and Khaled Comics (2020).
- Harry Potter's fandom speculated about the necessary death of Harry Potter (or one of his friends) to end Voldemort.
- For a long time, there were theories related to the different *ships* (relationships) of various characters in J. K. Rowling's saga.

### Explanatory Theories

The second block of theories includes interpretative proposals that attempt to cover hermeneutical gaps or gaps in the text. Explanatory theories are canonical interpretations that endow and (over) subscribe meaning to the events of the available canon. While predictive theories aim to infer outcomes explicitly, explanatory theories detail the complex process before the outcome. These semiotic mechanisms favor these interpretative proposals and explore the subtext, nuance, or implicit content linked to characters, functions, and narrative actions. In this sense, the theories that analyze the true motivations of the King of the Night and Voldemort are noteworthy.

Explanatory theories are found in specialized affinity spaces such as specific sections of *Reddit, Westeros*, the *lossietereinos, Archive Of Our Own* (*AO3*), and more recently, on specialized YouTube channels. In this sense, influencers in *YouTube* such as *Javi Marcos* are popular for their meticulous analysis and fan theories such as the one entitled *Why is Jon Snow's real name Aegon Targaryen also in the books* (Marcos, 2018) or Capa Invisible with videos such as *Theories of the founders of Hogwarts* (Capa Invisible, 2020). In these scenarios, complex theories and their accompanying evidence are presented and discussed through fragments from all TNs. They are collective constructions created by forensic fandom and readers with a highly developed sense of literary reading competence that show great expertise in recognizing and gathering clues that help to understand what has not been explicitly narrated. The fandom carries out original research, collection, documentation, and analysis of works.

Hence, in addition to all the official components of transmedia narratives, epitextual elements are fundamental for the development of these hermeneutical proposals: interviews with authors, statements on social networks, rumors, and leaks are some of the aspects that keep the fandom (and semiosis) active among publications, releases or broadcasts. Eco (1993, 1997) stated that empirical readers, in the form of a community, develop interpretative hypotheses that reconstruct the narrative, taking into account the author's intention or author-model.

They are not necessarily correct (they are hypotheses), but they are possible, canonical, and convincing. They provide answers to essential questions about the plot and justify and use narrative arguments to support a theory. They are accompanied by precise references to canonical elements: specific pages or fragments of the literary sagas, concrete frames in the audiovisual version, and epitextual elements. To this, we must add that collective intelligence feeds back and reinforces (or refutes) fan theories. In this regard, G. R. R. Martin notes:

The Internet affects all this to a degree it was never affected before […] Like Jon Snow's parentage. There were early hints about it in the books, but only one reader in 100 put it together. And before the Internet that was fine—for 99 readers out of 100 when Jon Snow's parentage gets revealed, it would be, “Oh, that's a great twist!” But in the age of the Internet, even if only one person in 100 figures it out then that one person posts it online and the other 99 people read it and go, “Oh, that makes sense” (Hibberd, 2019, par. 2).

Among the most outstanding examples we can note:

- The theory of R+L=J was raised in 1997 when only the first book was published. It has been one of the most widespread fandom theories and was confirmed in the canon almost 10 years later. This theory gives meaning to many details of the plot.
- Tyrion is the third head and, therefore, is Targaryen. Many events lead to this theory. We can find countless fragments and scenes used as a basis for this theory on the Internet.
- Dumbledore represents Death in *The Fable of the Three Brothers*, an interpretation of the fable that the author has confirmed on social networks (Rowling, 2015).

### Alternative Theories

Alternative theories are the least common and the most complex, as they offer unique, personal, improbable, and creative visions of the fictional universe. These interpretative proposals defy the limits of interpretation.

Structurally and narratively, they are similar to explanatory theories: they cover argumentative gaps and propose well-founded explanations. However, they veer sharply away from the shared interpretation of the community (fanon). The fandom would automatically discard these theories if it were not for the solid arguments that accompany them. It is precisely one of its main characteristics. Given the impact and breakdown of the horizon of expectations they cause, they need to be duly justified with explicit evidence to soften the proposals' implausibility. Despite this, they are understood as amusing theories but are not deemed viable interpretations by other users.

They often display a high level of formulation, creativity, and complexity. In this way, they allow readers with low literary and literary skills, through social reading, to achieve a greater textual understanding. What is relevant is that forensic fans exclusively create them, that is, those who dissect the canon and gather different fragments to construct the theory and persuade other users that such interpretations are viable, acceptable, and reliable.

Alternative theories comprise a high creative component and, therefore, usually have recognized authorship or source. Unlike other *fanworks* such as *fanfics* (transformative works which are character-centric), these readings keep the characters, the fictitious world in which they are placed, and the story narrated in the canon intact without including new narrative elements. While it is true that they update any potential meanings and interpretations of the text and are often overloaded with content, they are still suggested readings.

These theories are formed in specialized affinity spaces, but their playful character and interpretive shock value transcend and are subsequently published by other media and websites with more varied content. Most theories are timeless and current (even though new content has been published later): Could they be confirmed in the future? Yes, all alternative theories are possible, albeit improbable. From this perspective, alternative theories can pose new readings and can appear long after the narrative is completed. Some notable examples:

- Hogwarts is, in reality, a mental health institution in which Harry Potter is admitted, and the whole saga is a product of his imagination (see Roning_Ikari, 2016).
- Ron Wesley and Albus Dumbledore are actually the same character who has traveled in time with the aid of a Time-Turner to end Voldemort (later known as *Ronbledore*). Knight2King raised this theory in late 2003 (see Knight2king, 2004; Mallori, 2014). This particular reading has been maintained over time, and new arguments have been incorporated. However, 10 years later, the author, J. K. Rowling, intervened to refute this theory as it had garnered great relevance in the fandom (Rowling, 2015).
- Ned Stark is still alive. Different theories support this thesis:
  ◦ Eddard Stark possesses shifter powers (like his son). Before being executed, he changed his body, specifically to that of the birds flying away.
  ◦ Ned shared a cell with Jaqen (Syrian Forel), who could change his face. It was the latter who was executed. This would resignify Catelyn's surprise on seeing that Ned's bones were too small.
- The whole game of thrones responds to an evil plan of the Three-Eyed Raven and Brandon Stark (Faillace, 2019). This theory has inspired different *fanfics* and alternative endings (see, for example, Khaled Comics, 2020).
- Hagrid is actually a *Death Eater* (see Whoofph, 2019).

Below, Table 1 schematically develops the characteristics of each type of theory.

**Table 1:** Summary of analysis of fan theories.

	**Objective**	**Arguments**	**Elaboration**	**Adequacy**	**Impact and obsolescence**
Predictive	Infers narrative elements	No significant arguments	Individual	Canonical	Of little relevance to the fandom. Very abundant. Soon to be confirmed or refuted
Explanatory	Provides meaning to narrative events	Solid arguments with precise references to canonical elements	Fandom		The most transcendental theories. Expand the horizon of expectations of the fandom
Alternative	Rethinks reading and offers an alternate view		Individual	Non-canonical	Complex, unique, and peripheral. They are neither confirmed nor refuted by the canon

As vernacular literary practices, these three interpretative models are developed by highly motivated and committed users. Their participation in the processes of negotiation of meaning is mainly determined by the need to belong and be recognized in their affinity space and generate emotions linked to the canon or canonical text (Marina, 2007, 2011; Pink, 2010). This finding is highly relevant when transferring the proposal to the dynamics of formal education and, in particular, to “interpretive community” classes.

## Discussion

The Internet promotes the creation of virtual reading communities within which norms of production and reception of texts are negotiated and agreed on. The horizon of expectations common to its members is outlined: a reference system that guides them in reading and that translates into the activation of their previous experiences and knowledge about narrative sub-genres, formal, thematic, and discursive traits, as well as their ideas about what is literary (and transmedia) language as opposed to everyday or functional language.

As Culler (2004: p.80) stated, the interpretation of a work is developed to answer the questions formulated by this horizon of expectations. It is one in which the virtual reading community determines the types of valid or possible answers. In this case, the key lies in how textual details are used to link them to those answers. Fan theories, from this perspective, are forms of reading inscribed in the hermeneutic mechanisms of the community.

Fan theories are interpretive hypotheses proposed by empirical readers who collaborate and debate with other readers about the signs and clues that appear in the appellative structure of the text. Even when they occasionally successfully resolve certain plot unknowns ahead of the official narrative, the fandom does not reject these theories or consider them spoilers since they usually do not incorporate insider information. Given the large number and variety of interpretative possibilities offered, individual readers only access readings from other users and blur the lines of what is considered a spoiler, rumor (or hoax), fan theories, and *fanfics*. It enables readers in fan communities with low reading and literary competence to understand and access more profound levels of interpretation assisted by other followers in the community that helps to broaden their members' expectations horizon. These results match those of Ellithorpe and Brookes (2018).

Therefore, rather than being spoilers, these theories are an integrated and inherent part of the process of receiving (transmedia) analyzed narratives: readers enjoy the text individually and subsequently develop their own readings; adjusting and contrasting their interpretations with the community (*fanon*) (Jenkins, 2009; Jenkins et al., 2015; Völcker, 2017). Users thus carry out negotiated and consensual re-readings in order to understand and maintain an active semiosis.

Readers are often reluctant to say goodbye to their favorite texts and, to avoid doing so, participate in new social and creative activities that allow them to continue enjoying the plot or developing their favorite characters. From this point of view, the limits of interpretation are sometimes intentionally playfully outstripped with what Eco (1993, p. 86) calls “an aesthetic of the free, aberrant, intentional and malicious use of texts.” In this way, manifestly aberrant interpretations are shared. Fan theories are located in the semiotic debates of delimitation of these interpretative limits established by fandom, that is, the fanon. They generate extensive hermeneutical discussions within fan communities, as evinced by the research by Chaney and Liebler (2007) and Thomas (2018).

Alternative theories result from exhaustive textual analysis by readers with a developed literary competence in which information is collected and reconstructed in exceptionally creative ways: an invitation to rereading and reinterpret the text (Scolari et al., 2018).

However, it is pertinent to note that fan theories are only operational and functional (except for alternative theories, which are timeless) when the narrative is serialized and not concluded: they are formed, enjoyed, and discussed in the breaks between distributions of plot content. Generally speaking, if an asynchronous reading is performed (that is to say, approaching the text in a chronological period separate from the processes of cultural dissemination) or if it occurs after the saga has ended, fan theories are not relevant to the readership.

The hypothesis is that fan theories influence the artificial generation of expectations (hype) by overvaluing narrative possibilities to TN viewers and readers. Confirmation of these hypotheses could explain the rejection of the fandom to the outcomes of the main transmedia narratives: *Game of Thrones, Harry Potter, Star Wars*, or *Lost*.

## Data Availability Statement

The raw data supporting the conclusions of this article will be made available by the authors, without undue reservation.

## Author Contributions

All authors listed have made a substantial, direct and intellectual contribution to the work, and approved it for publication.

## Conflict of Interest

The authors declare that the research was conducted in the absence of any commercial or financial relationships that could be construed as a potential conflict of interest.
